# Patient and Family Satisfaction in the Intensive Care Unit of a Quaternary Care Center

**DOI:** 10.7759/cureus.45795

**Published:** 2023-09-22

**Authors:** Saleem Sharieff, Ayesha Sajjal, Asim Idrees, Wajid Rafai

**Affiliations:** 1 Intensive Care Unit, Pakistan Kidney and Liver Institute and Research Center, Lahore, PAK; 2 Intensive Care Unit, Grand River Hospital, Kitchener, CAN; 3 Critical Care Medicine, Pakistan Kidney and Liver Institute and Research Center, Lahore, PAK

**Keywords:** patients’ satisfaction, intensive care unit (icu), family satisfaction, survey, quality-control

## Abstract

Patient and family satisfaction is an indicator of quality assessment of care provided in the intensive care unit (ICU) ensuring that the quality of services provided meets not only the patients’ but also their families’ needs. Investigating how different variables affect their satisfaction ratings is important. We assessed patient and family satisfaction in a quaternary care center in Pakistan.

Methods: The study was a cross-sectional survey of adult patients and families treated between December 1, 2022 and April 30, 2023 in the ICU at Pakistan Kidney and Liver Institute and Research Center (PKLI-RC), Lahore, Pakistan. We used family satisfaction in ICU 24 (FS-ICU 24) to measure satisfaction in a number of domains on a scale of 1-5 (1 = Very Dissatisfied, 5 = Fully Satisfied).

Results: Of the 330 patients admitted to ICU during the study period, all patients and/or one of their family members (100%) participated in the study. Out of these, 209 (63%) were male. The mean age was 42 ± 15 years, and the overall mean patient and family satisfaction scores were 4.69 ± 0.69 and 4.55 ± 0.52, respectively. The mean score in all domains was > 4, with the exception of pain management in which it was 3.98 ± 0.53.

Conclusion: Patients and their families’ satisfaction is an important measure of ICU quality. Not only the patients and their families were satisfied with our ICU quality of care but they also appreciated involvement in the decision-making process and quality assessment. There is a need for further research for improvement in the pain domain.

## Introduction

Patient and family satisfaction from care provided in hospitals and intensive care units (ICU) is widely used to assess the quality of care. The patient and their family are anxious about the patient’s condition, ICU procedures, and risk of complications, prognosis, and other unfamiliar experiences when admitted to ICU. Poor satisfaction is largely due to the gap between expectations from services provided and the consummation of perceived needs that may subsequently affect patient outcomes [1]. Providing patients and families information about patients’ conditions and outcomes in the ICU may help lower anxiety and frustration and increase confidence, trust, and satisfaction with the healthcare team.

Examining the perspectives of patients and their family members and implementing strategies to address areas of concern is essential to improve the ICU care quality. Previous studies have focused separately on satisfaction with general ICU care, preoperative psychological distress, and informational needs related to surgical care but no such study has been reported from Pakistan [2-4]. Thus, we conducted this study to assess patients’ and their families’ satisfaction with ICU services in our institute.

## Materials and methods

This study was a cross-sectional survey conducted in the ICU of Pakistan Kidney and Liver Institute and Research Center (PKLI-RC), Lahore, Pakistan. ICU patients and/or one of their family member participated in the study survey. PKLI-RC is a 250-bedded quaternary care center. It has an ICU with 14 surgical, eight medical, and four pediatric beds with 24-hour intensivist coverage. Eligibility criteria included all adult patients treated between December 1, 2022 and April 30, 2023.

Next, we used family satisfaction in ICU 24 (FS-ICU 24) to measure satisfaction in a number of domains on a scale of 1-5 (1 = Very Dissatisfied, 5 = Fully Satisfied). This questionnaire is already validated and has been widely used [5-9]. To use it in our setting, we translated it into Urdu language using Google translator (https://translate.google.com/?sl=en&tl=ur&op=translate). We pilot-tested it on non-study participants for face validity and rephrased some of the questions for easy understanding of the participants.

To assess full ICU experience, a questionnaire was completed at the time of ICU discharge or after the patient’s death. In case of death, the next of kin completed the questionnaire. Participants’ demographic data were collected that included age, gender, and relationship with the patient. We also collected information on survival status at the time of ICU discharge. The study coordinator, who had no direct responsibility for patient care, completed the questionnaire. Data analyses were conducted using SPSS 20 software (IBM). Descriptive statistics (means, standard deviations, and proportions) were used to summarize the data. The institutional review board (IRB) of the PKLI hospital (PKLI-IRB/AP/135) approved the study.

## Results

A total of 330 adult patients were admitted to ICU between December 1, 2022 and April 30, 2023. All patients or their next of kin (100%) completed the questionnaire. Of the 330 patients, 209 (63%) were male. Mean age of the whole cohort was 42 ± 15 years, and 288 (87%) were alive at the time of ICU discharge (Table 1).

**Table 1:** Participants’ demographic characteristics. ^a^x̄ ± SD; all such values ^b^Median (min - max) ^c^n (%); all such values

Patients, N	330
Age (Years)	42 ± 15^a^
Length of ICU stay (Days)	2 (1 - 32)^b^
Male, n (%)	209 (63.3)^c^
Out of town residence, n (%)	238 (72)
Alive at ICU discharge, n (%)	288 (87)
Next of Kin, N	330
Age (years)	36 ± 9
Relationship	
Spouse, n (%)	56 (17)
Sibling, n (%)	112 (34)
Children, n (%)	94 (29)
Parent, n (%)	44 (13)
Other relatives, n (%)	24 (7)

Table 2 shows responses collected from 288 patients who were alive at the time of hospital discharge; 42 (13%) patients had died. The highest satisfaction score was given to the ICU doctors’ skills and competency, followed by consultants’ support during depression and communication. Patients were less satisfied with their met needs per expectations, the ICU’s pain control, and the level of emotional support provided. Table 3 shows responses from 330 family members of patients to our questionnaire. The highest score was given to satisfaction with consultants’ care, followed by ICU doctors’ skill and competency, and the ICU team’s communication with family. Patients and families largely answered “very satisfied” to each question in both categories. The overall mean patient and family satisfaction scores were 4.69 ± 0.69 and 4.55 ± 0.52, respectively. The mean score in all domains was > 4, with the exception of pain management in which it was 3.98 ± 0.53.

**Table 2:** Patients’ satisfaction survey. ^a^n (%); all such values. ^b^x̄ ± SD; all such values. Note: Satisfaction score is on a scale of 1-5 (1 = Very Dissatisfied, 5 = Fully Satisfied). N/A: Not assessed are those patients who were unable to answer questionnaires due to sickness or death).

Variables	Very Dissatisfied, n (%)	Slightly Dissatisfied, n (%)	Mostly Satisfied, n (%)	Very Satisfied, n (%)	Completely satisfied, n (%)	N/A, n (%)	Mean (±SD)
A: How did we treat your symptoms?							
Concern and caring	0	1 (0.3)^a^	16 (4.8)	185 (56.1)	86 (26.1)	42 (12.7)	4.24 ± 0.58^b^
Pain	0	4 (1.2)	44 (13.3)	211 (63.9)	29 (8.8)	42 (12.7)	4.19±0.87
Breathlessness	0	0	38 (11.5)	198 (60)	52 (15.8)	42 (12.7)	4.31±0.84
Agitation/delirium	0	0	44 (13.3)	188 (57)	56 (17)	42 (12.7)	4.29±0.86
Anxiety	0	0	50 (15.2)	187 (56.7)	51 (15.4)	42 (12.7)	4.26±0.87
Depression	0	1 (0.3)	39 (11.8)	189 (57.3)	59 (17.9)	42 (12.7)	4.31±0.86
B: How much did we support you?							
Showed interest in your needs	0	0	21 (6.4)	188 (57)	79 (23.9)	42 (12.7)	4.16±0.58
Emotional support	0	0	19 (5.8)	182 (55.2)	87 (26.4)	42 (12.7)	4.21±0.57
Co-ordination of care	0	0	13 (3.9)	183 (55.5)	92 (27.9)	42 (12.7)	4.23±0.57
Courtesy, respect, and compassion	0	0	14 (4.2)	177 (53.6)	97 (29.4)	42 (12.7)	4.25±0.58
Support during depression	0	0	19 (5.8)	206 (62.4)	63 (19.1)	42 (12.7)	4.39±0.79
Support during anxiety	0	0	27 (8.2)	208 (63)	53 (16.1)	42 (12.7)	4.34±0.81
C: How satisfied are you with ICU staff including doctors, nurses, and allied health?							
Skills and competency of ICU staff	0	0	13 (3.9)	180 (54.5)	95 (28.8)	42 (12.7)	4.28±0.54
Communication with ICU staff	0	0	13 (3.9)	163 (49.4)	112 (33.9)	42 (12.7)	4.35±0.56
Skills and competence of ICU doctors	0	0	9 (2.7)	150 (45.5)	129 (39.1)	42 (12.7)	4.41±0.56
Are you satisfied with ICU consultants?	0	0	8 (2.4)	160 (48.5)	120 (36.4)	42 (12.7)	4.37±0.54
D: How was overall ICU care? Your experience and satisfaction with care provided in ICU.							
Were goals of care met?	0	0	9 (2.7)	172 (52.1)	107 (37.4)	42 (12.7)	4.33±0.54
Understanding of information	0	0	12 (3.6)	149 (45.2)	127 (38.5)	42 (12.7)	4.31±0.57
Support provided during decision-making process	0	0	2 (0.6)	199 (60.3)	88 (26.7)	41 (12.4)	4.28±0.47
Overall satisfaction	0	0	2 (0.6)	141 (42.7)	145 (43.9)	42 (12.7)	4.46±0.52

**Table 3:** Patients’ families’ satisfaction survey. ^a^n (%); all such values. ^b^x̄ ± SD; all such values. Note: Satisfaction score is on a scale of 1-5 (1 = Very Dissatisfied, 5 = Fully Satisfied).

Variables	Very Dissatisfied, n (%)	Slightly Dissatisfied, n (%)	Mostly Satisfied, n (%)	Very Satisfied, n (%)	Completely satisfied, n (%)	Mean (±SD)
A: How did we treat your patient’s symptoms?						
Concern and caring by ICU staff	0	0	22 (6.7)^a^	216 (65.5)	92 (27.9)	4.21±0.55^b^
Pain	0	1 (0.3)	45 (13.6)	242 (73.3)	42 (12.7)	3.98±0.53
Breathlessness	0	1 (0.3)	35 (10.6)	208 (63)	86 (26.1)	4.15±0.59
Agitation/delirium	0	0	40 (12.1)	214 (67.3)	69 (21.7)	4.09±0.57
Anxiety	0	1 (0.3)	54 (16.4)	212 (64.2)	63 (19.1)	4.02±0.61
Depression	0	1 (0.3)	50 (14.8)	212 (64.2)	67 (20.3)	4.04±0.61
B: How much did we support you?						
Did ICU staff show interest in your needs?	0	0	24 (6.4)	202 (61.2)	107 (32.4)	4.26±0.57
Emotional support	0	0	17 (5.2)	200 (60.6)	113 (34.2)	4.29±0.56
Co-ordination with ICU staff	0	0	15 (4.5)	195 (59.1)	120 (36.4)	4.32±0.56
Was courtesy, respect, and compassionate care provided?	0	0	17 (5.2)	186 (56.4)	127 (38.5)	4.33±0.57
Support during depression	0	1 (0.3)	26 (7.9)	210 (63.6)	93 (28.2)	4.19±0.58
Support during anxiety	0	0	39 (11.8)	216 (65.5)	75 (22.7)	4.11±0.58
C: How satisfied are you with ICU staff including doctors, nurses, and allied health?						
Skills and competency of ICU staff	0	1 (0.3)	16 (4.8)	175 (53)	138 (41.8)	4.36±0.59
Communication with ICU staff	0	0	12 (3.6)	168 (50.9)	150 (45.5)	4.42±0.56
Skill and competency of ICU Doctors	0	0	11 (3.3)	150 (45.5)	169 (51.2)	4.48±0.56
Are you satisfied with ICU consultant?	0	0	8 (2.4)	145 (43.9)	177 (53.6)	4.51±0.55
D: How was overall ICU care? Your experience in ICU?						
Were goals of care met?	0	1 (0.3)	12 (3.6)	193 (58.5)	124 (37.6)	4.33±0.58
Understanding of information	0	0	12 (3.6)	171 (51.8)	147 (44.5)	4.41±0.56
Support provided during decision-making process	0	0	0	195 (59.1)	135 (40.9)	4.41±0.49
Family’s overall satisfaction	0	0	4 (1.2)	141 (42.7)	185 (56.1)	4.55±0.52

## Discussion

There are many stages and trajectories that can occur during a critical illness, such as organ malfunction, deterioration, recovery, and death. In addition to being psychologically, physically, and emotionally upsetting, these difficulties endured by patients and their families during ICU stays also cause significant psychological anguish. Patients and their families might feel more involved in and connected to the ICU experience with good communication. This is the first patient and family satisfaction survey reported from an ICU in Pakistan. The survey provides information about patients’ and their families’ experiences with ICU care quality. We found that patients and their families positively responded to the call for participation in the study survey. The response rate was 100% and the overall satisfaction score reflected that the patients and families were “very satisfied”. There was a similar pattern in scores across all domains except for pain management, where the score reflected that patients and families were “somewhat satisfied”.

To measure family happiness, Dodek et al. considered quality improvements as the primary objective [10]. Although there are many general satisfaction measures in literature, they typically focus on areas such as staff empathy and attitude toward patients, access to and compliance with caregivers, or technical competencies. Access to information on patient care and the quality of relationships among patients and family members as essential factors for happiness are equally important. Therefore, surveys that are specific and multi-dimensionally covering all components of ICUs should be developed to measure satisfaction, which may be influenced by a range of factors.

Four key satisfaction domains have been addressed by our study, which are related to symptom management, support made available, knowledge and competence of the ICU team, communication, and involvement with decision-making processes as these instruments represent an essential element for measurement of total satisfaction. The importance of communication has been emphasized by numerous studies as it reflects the attitude, behavior, empathy toward patients and their families, and competency of the physician [8,11-15]. Longer communication periods between healthcare providers and families were found to be associated with reduced anxiety among ICU patients’ family members [14]. We found that communication with patient and their families by the medical team helps them in understanding and tracking medical information which ultimately helps relieve distress related to critical illnesses. Multidisciplinary communication strategy incorporating five objectives described in the mnemonic “VALUE” (to value and appreciate what the family members said, to acknowledge the family members’ emotions, to listen, to ask open-ended questions that would allow the caregiver to understand who the patient was as a person, and to elicit questions from family members) was shown to lessen symptoms of anxiety, depression, and post-traumatic stress disorder [15]. Family satisfaction in the ICU also increased through training and teaching communication skills, including listening capacity and opportunities for families to share information at conferences [16]. The ICU environment has been identified as a factor that affects satisfaction and our study population also commented on hospital facilities in waiting areas and advised improvement [8,17].

Based on the items in this survey, it is not easy to measure overall satisfaction with a composite score or a latent construct. However, the areas with the highest scores were ICU doctors’ skills and competency, followed by consultants’ support during depression and care. The areas requiring improvement were patients’ needs, including controlling symptoms such as pain and providing more emotional support. While patients’ families showed satisfaction toward ICU consultants and the emotional support provided, families sought more support for patients’ symptom control and help with their own depression and anxiety in critical situations. These results are consistent with prior studies [17,18].

The aggressiveness of the treatment a patient wishes to take and their level of involvement in decisions varies among patients and their families. Our study’s aim was to ascertain to what extent patients and families were comfortable with the health care’s aggressiveness and how much they participated in decision-making related to care. We found that structured and scheduled family meetings with proper communication with patients and families lead to better satisfaction with the care provided, as has been proven in other studies [19].

The fact that this work provides a tool to evaluate the quality of care provided based on input from patients and their families, even that of deceased patients, is one of its merits. Second, the high survey response rate proved the viability of this questionnaire in both English and Urdu. Despite the fact that it was a single-center study, no causal relationship could be established and conclusions could not be generalized outside of the ICU or to other institutions. Nevertheless, this assessment is a unique experience from a large ICU in Pakistan and showed high satisfaction scores in most items; however, there is always room for further improvement. Furthermore, it has been found that the degree of satisfaction may significantly affect family members’ relationships with ICU patients and their outcomes.

## Conclusions

Patients’ and their families’ satisfaction is important for quality assessment. In our study, patients and their families were largely satisfied with the care provided during their ICU stay, their involvement in decision-making, and assessment for quality control through this survey. This demonstrates the value of the critical care team communicating with patients and their families and involving them in the care plan, including the goals of treatment. Our results are comparable with most overseas centers, however, there still remains room for improving the ICU environment, delirium management, and further improvement in communication with families. This study provides vital information to hospital management and other political decision-makers to enhance the quality of care and service development.
